# The impact of national centralized drug procurement on health expenditures for lung cancer inpatients: A difference-in-differences analysis in a large tertiary hospital in China

**DOI:** 10.3389/fpubh.2022.956823

**Published:** 2022-08-12

**Authors:** Yuan-jin Zhang, Yan Ren, Quan Zheng, Jing Tan, Ming-hong Yao, Yun-xiang Huang, Xia Zhang, Kang Zou, Shao-yang Zhao, Xin Sun

**Affiliations:** ^1^Chinese Evidence-Based Medicine Center, West China Hospital, Sichuan University, Chengdu, China; ^2^NMPA Key Laboratory for Real World Data Research and Evaluation in Hainan, West China Hospital, Sichuan University, Chengdu, China; ^3^Sichuan Center of Technology Innovation for Real World Data, West China Hospital, Sichuan University, Chengdu, China; ^4^Hainan Healthcare Security Administration Key Laboratory for Real World Data Research, West China Hospital, Sichuan University, Chengdu, China; ^5^Department of Thoracic Surgery and Institute of Thoracic Oncology, West China Hospital, Sichuan University, Chengdu, China; ^6^Department of Finance, School of Economics, Sichuan University, Chengdu, China

**Keywords:** cancer, drug policy, evidence-based policy, health economics, policy evaluation

## Abstract

The availability and affordability of medicines remain major health challenges around the world. In March 2019, the Chinese government introduced a pilot National Centralized Drug Procurement (NCDP) program in order to reduce drug prices and improve the affordability of effective and safe medicines. This study aimed to assess the impact of NCDP policy on health expenditures of cancer patients. Using inpatient discharge records from a large hospital in the pilot city, we performed a difference-in-differences design to estimate the change in health expenditures before and after the policy. We found that the implementation of NCDP was associated with a significant decrease in total expenditures (14.13%) and drug expenditures (20.75%) per inpatient admission. There were also significant reductions in non-drug-related expenditures, including a 7.65% decrease in health service expenditures, a 38.28% decrease in diagnosis expenditures, and a 25.31% decrease in consumable material expenditures per inpatient admission. However, the NCDP implementation was associated with a 107.97% increase in the traditional Chinese medicine expenditures. Overall, the study provided evidence that the NCDP policy has achieved its goals of high-quality and affordable healthcare. The drug expenditures of lung cancer patients revealed a continuous decline, and the policy may have spillover effects on other healthcare expenditures. Further studies are needed to evaluate the long-term effects of NCDP on policy-related expenditures and health outcomes.

## Introduction

The affordability and availability of medicines remain the major issues for healthcare systems globally, especially for patients in developing countries (1–5). Despite a series of drug policies being implemented since the major healthcare reform in China from 2009, such as the National Essential Medicine Policy and the Zero Mark-up Drug Policy, drug expenditures are still increasing every year and the rising drug expenditures have been serious burdens for both family and society in China (6, 7).

To reduce drug expenditures of patients, in March 2019, China launched the National Centralized Drug Procurement (NCDP) program. There were 11 cities selected as the first round of NCDP pilot cities, including four municipalities (Beijing, Tianjin, Shanghai, and Chongqing) and seven major cities (Shenyang, Dalian, Xiamen, Guangzhou, Shenzhen, Chengdu, and Xi'an); thus, the policy was also known as the “4+7” policy. As a major reform of the current drug procurement system, the NCDP requested all public hospitals in the pilot cities to purchase 60 to 70% of their total annual demand for selected drugs, which aimed to achieve a lower price in exchange for a larger volume of purchase (8).

Anticancer drugs account for the highest proportion of pharmaceutical spending among all therapeutic classes in China, and the heavy economic burden on cancer patients attracted the attention of policymakers (9–13). In the first round of NCDP, Pemetrexed and Gefitinib, two anticancer drugs for the first-line lung cancer treatment were included, which were the two most expensive drugs in the procurement list and had prices cut by 71 and 76%, respectively (5). Unlike drugs for chronic disease in the NCDP program, anticancer drugs are often used in combination with other treatments (14). Previous studies found that, despite the fact that drug price decreased after the policy (e.g., the Drug Zero Mark-up policy), there were no measurable changes in total expenditures, as the expenditures for diagnostic tests and medical consumables were increased (15). Therefore, whether the decline in drug prices can reduce the economic burden on patients after the NCDP remains to be further verified.

Although previous studies have reported the potential impact of the NCDP policy on drug expenditures, none of them focused on anticancer drugs. Most studies found that the volume of policy-related drugs increased, while the purchase spending declined after the implementation of NCDP (4, 16, 17). The policy effects on antihypertensive drugs, antibiotic drugs, and nucleoside analogs were consistent with the overall policy effects (18–20).

Additionally, the data of most previous studies were pharmaceutical procurement records and they evaluated the drug purchase spending at health facility level. Although there is strong consistency between drug purchase data and drug use data (such as prescriptions and claims), it is possible that the policy effects on patients could not be evaluated through procurement records (21, 22). Only one study used the hospital information system data, but this study applied an interrupted time series design and estimated the average monthly drug expenditures of patients treated in outpatient and emergency departments (23).

In this study, we used inpatient discharge records from a large oncology specialized hospital in Chengdu, one of the pilot cities, and adopted a difference-in-differences (DID) approach to evaluate the impact of NCDP policy on the health expenditures of lung cancer inpatients. Using individual-level data and a quasi-experimental design, our study added strong patient-level evidence to comprehensively reflect the policy effects on expenditures of patients during hospitalization.

## Materials and methods

### Study design

We performed a DID design to estimate the NCDP policy effects, which is a popular study design to compare outcomes before and after a policy change for one group affected by the policy (treatment group) and another group not affected by the policy (control group) (24, 25). As a strong quasi-experimental design to mimic the experimental design, DID analysis is much better than traditional observational studies of controlling only for observed confounding *via* regression modeling (26).

The two bid-winning products, Pemetrexed and Gefitinib, were recommended for the first-line chemotherapy and targeted therapy for the treatment of non-small cell lung cancer (NSCLC). Thus, the treatment group of DID design included the patients with lung cancer who mainly received chemotherapy and targeted therapy during the hospitalization. And the control group included the patients with other types of cancer who received chemotherapy and targeted therapy, for whose expenditures were not affected by the NCDP policy.

### Setting and data source

Chengdu was one of the pilot cities in the first round of NCDP policy, and the policy was implemented on 25 March 2019. We used the data from a large tertiary-grade level-A oncology specialized hospital in southwest China, which had more than 400,000 outpatients and 60,000 inpatients annually and could be representative enough for the pilot city. The data were extracted from 1 January 2019 to 31 December 2019 from inpatient discharge records of the hospital, which contained the information of patients during hospitalization, including diagnosis, treatment, operation, expenditures, and payment way. Ethical approval for the study was not required because no potentially identifiable human data were used and presented in this study.

### Sample selection

Based on the International Classification of Diseases 10 (ICD-10) codes, we identified therapy type and cancer type of patients by primary diagnosis and secondary diagnosis, respectively. We included patients with the following criteria: (1) inpatient; (2) the patient was discharged from the hospital between 1 January 2019 and 31 December 2019; and (3) the main therapy (primary diagnosis) was chemotherapy or targeted therapy. Considering the representativeness of cancer patients, we excluded the patients with rare cancer (< 500 observations). Overall, a total of 23,443 cases were selected in our study, including patients with lung cancer, breast cancer, cervical cancer, ovarian cancer, colon cancer, rectal cancer, gastric cancer, non-follicular lymphoma, liver cancer, nasopharyngeal cancer, corpus uteri cancer, and esophageal cancer. The ICD codes for inclusion and exclusion criteria are summarized in Table 1.

**Table 1:** ICD-10 code for inclusion and exclusion criteria.

**Variable name**	**ICD-10 Code**	**Diagnosis**
Primary diagnosis	Z51.1	Chemotherapy session for neoplasm
	Z51.8	Other specified medical care (Target therapy)
Secondary diagnosis	C50	Malignant neoplasm of breast
	C53	Malignant neoplasm of cervix uteri
	C34	Malignant neoplasm of bronchus and lung
	C56	Malignant neoplasm of ovary
	C18	Malignant neoplasm of colon
	C20	Malignant neoplasm of rectum
	C16	Malignant neoplasm of stomach
	C83	Non-follicular lymphoma
	C22	Malignant neoplasm of liver and intrahepatic bile ducts
	C11	Malignant neoplasm of nasopharynx
	C54	Malignant neoplasm of corpus uteri
	C15	Malignant neoplasm of esophagus

### Outcome measurements

The outcomes were expenditures of cancer patients per hospitalization which contain the expenditures could be reimbursed or not, including total expenditures, drug expenditures (Western medicine), health service expenditures, diagnosis expenditures, treatment expenditures, consumable material expenditures, and traditional Chinese medicine (TCM) expenditures. Health service expenditures included general medical service fees, medical operation fees, nursing fees, and other health service fees. Treatment expenditures included surgical treatment fees and non-surgical treatment fees. Diagnosis expenditures included pathological diagnosis fees, laboratory diagnosis fees, imaging diagnosis fees, and clinical diagnosis fees.

### Statistical analysis

We described patient characteristics and outcomes stratified by groups and time. Patient characteristics included age, gender, metastasis, treatment type, payment way, and length of stay. To test the difference between the two groups, we used *t*-test for continuous variables and chi-square test for categorical variables.

Following the DID design, the impact of NCDP policy was estimated by comparing the differences between (1) changes between the pre- and post-intervention periods within the treatment group (patients with lung cancer) and (2) changes between the pre- and post-intervention periods within the control group (patients with other types of cancer). We applied the DID method using the following equation:


(1) $$\begin{array}{r} \begin{array}{c} \begin{array}{l} \log(Y_{it})=\beta_{0}+\beta_{1}Treat_{i}+\beta_{2}Time_{t}+\beta_{3}Treat_{i}*Time_{t}\;\\ \;+\beta \text{ Σ}Z_{it}+\delta_{t}+\varepsilon_{it}\; \end{array} \end{array} \end{array}$$

where *Y*_*it*_ refers to the expenditures of a patient *i* who was hospitalized in time *t*. *Treat*_*i*_ is a dummy variable that coded 1 for the lung cancer patient and 0 otherwise. *Time*_*t*_ is also a dummy variable that coded 0 before the NCDP policy and 1 after the policy. The vector *Z*_*it*_ is a vector of covariates to adjust for characteristics of patients. δ_*t*_ is a series of variables used to control monthly linear and quadratic time trends. We used ordinary least square with robust standard errors in DID regression. The DID estimation β_3_ is an interaction variable between *Treat*_*i*_ and *Time*_*t*_, which represents the effects of NCDP. Since the expenditures were not always normal distribution, all the expenditure data used in DID regression were expressed in the logarithmic form. Thus, the interpretation of β_3_ is the rate of change in log(Y) as X varies. The percentage change in Y as X varies could be calculated as 100($e^{\;\beta_{3}\;}$-1)%, which directly reflected the effects of NCDP on expenditures (27). There were no missing data in the dataset.

An important assumption of DID analysis is that there would be parallel trends in the outcomes between the treatment group and the control group in the absence of the NCDP policy. We tested the parallel trends assumption in two ways. First, we plotted the monthly trends of medical expenditures by the treatment group and the control group, respectively. Then, we implemented an event study approach that would more specifically trace out the timing of effects (28). The regression model was defined in the following equation:


(2) $$\begin{array}{r} Y_{it}=\beta_{0}+\sum_{j=-3,-2,1,2,3\ldots }\beta_{j}Treat_{i}*Month_{j}\\ \;+\text{β}\Sigma Z_{it}+\varepsilon_{it} \end{array}$$

where *Month*_*j*_ is a dummy variable that coded 1 if the patient was discharged from the hospital in the month j. And j means the month prior or post to the policy. We identified that April was the first month after the implementation of the NCDP (j = 1). Then, we constructed a series of dummies: 3 months before policy (j = −3), 2 months before policy (j = −2), 1 month after policy (j = 1), and 2 and more months after policy (j = 2, 3, …). The month just prior to policy (j = −1) was excluded as the reference. Other variables are the same as the equation (1). β_*j*_ represents the difference between the treatment group and the control group in the month j. The common trends assumption is appropriate if the coefficients before the policy are not statistically significant.

To explore the robustness of our main results, we carefully reviewed the clinical guidelines and policies related to cancer to see whether there are some significant changes in the study period. Then, we performed a series of sensitivity analyses to eliminate the potential influence of selection bias and confounding. First, we implemented a placebo test based on a series of randomized treatment groups. Second, we excluded breast cancer, cervical cancer, ovarian cancer, and corpus uteri cancer patients in the control group to avoid confounding by gender differences. Third, we plotted the raw data of our whole sample and found that the expenditures rapidly fell in November and rose in December. Considering that there might be some events at the end of the year (such as medical insurance settlement and hospital performance assessment), we excluded the patients in November 2019 for inconsistent trends of outcomes in the month (29). Finally, considering the potential seasonality in one-year time, we added Fourier terms to capture it. All statistical analyses were performed using Stata 15.1.

## Results

There were 27,412 inpatients with chemotherapy or targeted therapy in the dataset. We excluded patients with rare cancer (< 500 cases) and finally included 23,443 patients. Patients in the treatment group were lung cancer patients (*N* = 3,636), and patients in the control group were patients with other types of cancers (*N* = 19,807), including breast cancer (*N* = 6,511), cervical cancer (*N* = 3,980), ovarian cancer (*N* = 1,770), colon cancer (*N* = 1,452), rectal cancer (*N* = 1,372), gastric cancer (*N* = 1,304), non-follicular lymphoma cancer (*N* = 858), liver cancer (*N* = 699), nasopharyngeal cancer (*N* = 640), corpus uteri cancer (*N* = 612), and esophageal cancer (*N* = 609).

The descriptive statistics are summarized in Table 2. Baseline absolute differences in age, gender, metastasis, treatment type, payment way, and length of stay were statistically significant. These characteristics were controlled in the DID regression model.

**Table 2:** Baseline characteristics for cancer patients in treatment group and control group.

	**Treatment group**			**Control group**			**Difference (*p*-value)**
**Variables**	**Overall (*N* = 3,636)**	**Before NCDP (*N* = 839)**	**After NCDP (*N* = 2,797)**	**Overall (*N* = 19,807)**	**Before NCDP (*N* = 4,020)**	**After NCDP (*N* = 15,787)**	
**Age [Mean (SD)]**	58.63 (8.81)	57.33 (9.49)	59.02 (8.56)	52.64 (10.30)	52.70 (10.43)	52.63 (10.27)	<0.001
**Length of stay [Mean (SD)]**	6.24 (4.29)	6.91 (4.52)	6.03 (4.20)	5.12 (3.75)	5.62 (4.14)	4.99 (3.63)	<0.001
**Gender [*****N*** **(%)]**							
Male	2,594 (71.3%)	578 (68.9%)	2,016 (72.1%)	4,868 (24.6%)	1,069 (26.6%)	3,799 (24.1%)	<0.001
Female	1,042 (28.7%)	261 (31.1%)	781 (27.9%)	14,939 (75.4%)	2,951 (73.4%)	11,988 (75.9%)	
**Metastasis [*****N*** **(%)]**							<0.001
No	1,144 (31.5%)	317 (37.8%)	827 (29.6%)	15,264 (77.1%)	3,069 (76.3%)	12,195 (77.2%)	
Yes	2,492 (68.5%)	522 (62.2%)	1,970 (70.4%)	4,543 (22.9%)	951 (23.7%)	3,592 (22.8%)	
**Treatment type [*****N*** **(%)]**							<0.001
Maintenance chemotherapy for malignant tumors	2,445 (67.2%)	520 (62.0%)	1,925 (68.8%)	8,542 (43.1%)	1,595 (39.7%)	6,947 (44.0%)	
Chemotherapy of malignant tumors after surgery	984 (27.1%)	285 (34.0%)	699 (25.0%)	10,049 (50.7%)	2,254 (56.1%)	7,795 (49.4%)	
Targeted therapy for malignancies	109 (3.0%)	19 (2.3%)	90 (3.2%)	104 (0.5%)	22 (0.5%)	82 (0.5%)	
Chemotherapy of malignant tumors before surgery	12 (0.3%)	3 (0.4%)	9 (0.3%)	848 (4.3%)	140 (3.5%)	708 (4.5%)	
Other treatment types	86 (2.4%)	12 (1.4%)	74 (2.6%)	264 (1.3%)	9 (0.2%)	255 (1.6%)	
**Payment type [*****N*** **(%)]**							<0.001
Urban Employee Basic Medical Insurance	3,200 (88.0%)	751 (89.5%)	2,449 (87.6%)	17,551 (88.6%)	3,583 (89.1%)	13,968 (88.5%)	
Urban Resident Basic Medical Insurance	362 (10.0%)	82 (9.8%)	280 (10.0%)	1,562 (7.9%)	383 (9.5%)	1,179 (7.5%)	
New Cooperative Medical Scheme	5 (0.1%)	0 (0.0%)	5 (0.2%)	32 (0.2%)	8 (0.2%)	24 (0.2%)	
Other payment types	69 (1.9%)	6 (0.7%)	63 (2.3%)	662 (3.3%)	46 (1.1%)	616 (3.9%)	

(1) The overall difference between the treatment group and the control group.

### Trends for health expenditures of cancer patients

**Figure 1:**
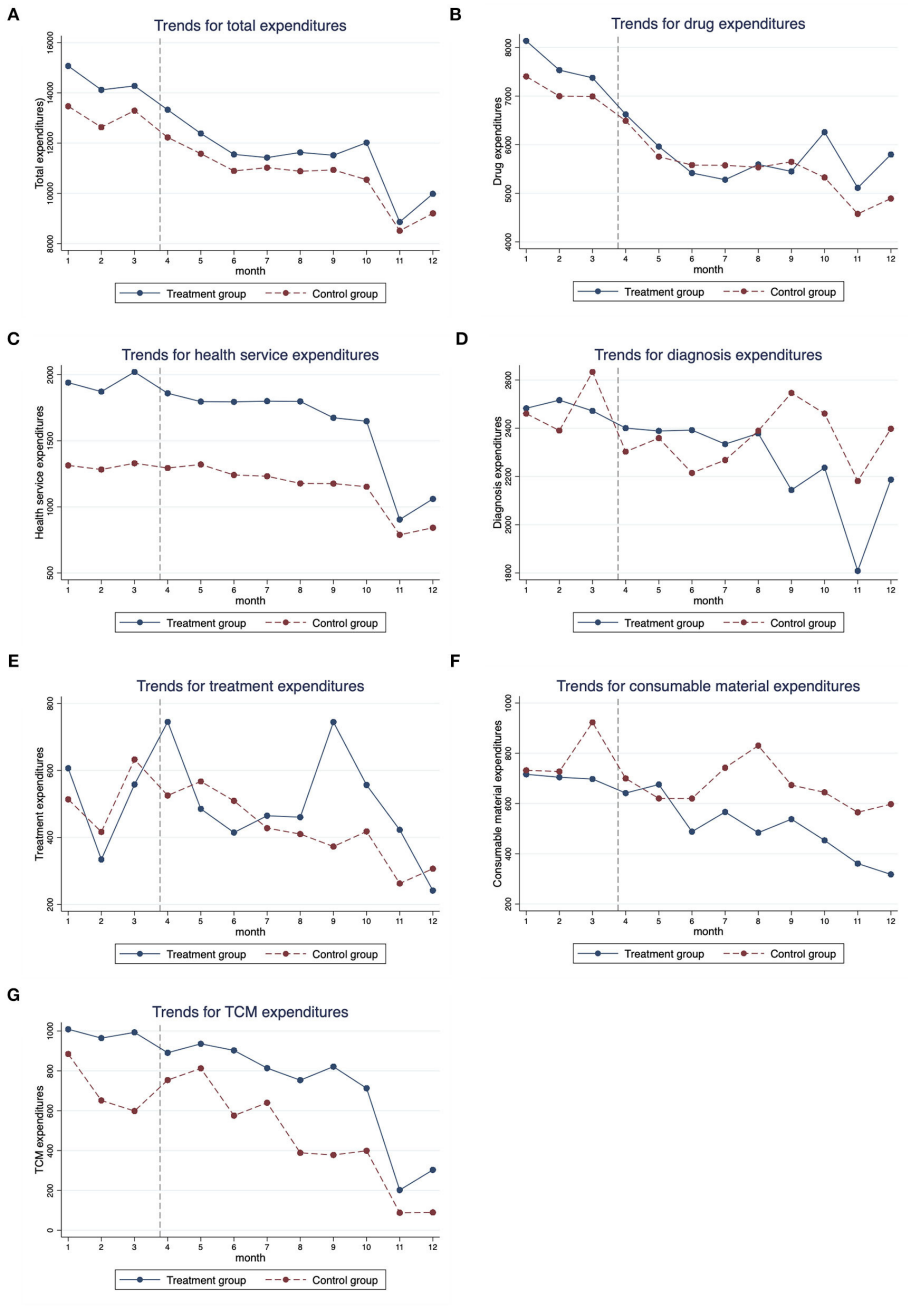
Monthly trends for medical expenditures of cancer patients from January 2019 to December 2019. **(A)** Total expenditures, **(B)** drug expenditures, **(C)** health service expenditures, **(D)** diagnosis expenditures, **(E)** treatment expenditures, **(F)** consumable material expenditures, and **(G)** TCM expenditures.

Table 3 reports the descriptive analysis of all outcomes and Figure 1 visualizes the trends in monthly health expenditures by the treatment group and the control group. The trends were similar for the two groups before the NCDP policy, indicating that the two groups were comparable. After the policy, trends in all types of expenditures declined in both the groups, but the changes were more notable in the treatment group. For example, the change of total expenditures for patients in the treatment group was −21.37% [the average total expenditures per hospitalization was 14,536.21 Chinese Yuan (CNY) before the policy and 11,429.45 CNY after the policy], and the change of total expenditures for patients in the control group was −19.17% (the average total expenditures per hospitalization was 13,166.93 CNY before the policy and 10,642.66 CNY after the policy).

**Table 3:** Expenditures of cancer patients and effects of the NCDP policy on medical expenditures (CNY).

	**Descriptive statistics (Mean)**						**DID estimation [**β **(95% CI)]**		**Effects of the NCDP 100(e^β^−1) %**
	**Treatment group**			**Control group**			**(1)**	**(2)**	
	**Before NCDP**	**After NCDP**	**Change**	**Before NCDP**	**After NCDP**	**Change**	**Unadjusted model**	**Adjusted model**	
Total expenditures	14,536.21	11,429.45	−21.37%	13,166.93	10,642.66	−19.17%	−0.1789^***^ (−0.2365, −0.1214)	−0.1523^***^ (−0.2006, −0.1040)	−14.13%
Drug expenditures	7,715.74	5,704.04	−26.07%	7,147.97	5,485.11	−23.26%	−0.2633^***^ (−0.3589, −0.1676)	−0.2326^***^ (−0.3219, −0.1432)	−20.75%
Health service expenditures	1,946.45	1,607.89	−17.39%	1,310.30	1,135.49	−13.34%	−0.1186^***^ (−0.1777, −0.0595)	−0.0796^***^ (−0.1177, −0.0414)	−7.65%
Diagnosis expenditures	2,488.98	2,261.07	−9.16%	2,499.16	2,351.08	−5.93%	−0.5338^***^ (−0.6361, −0.4315)	−0.4826^***^ (−0.5749, −0.3903)	−38.28%
Treatment expenditures	511.79	502.54	−1.81%	526.08	419.56	−20.25%	−0.1220^*^ (−0.2252, −0.0188)	−0.0476 (−0.1410, 0.0459)	−4.65%
Consumable material expenditures	706.86	505.52	−28.48%	795.68	668.91	−15.93%	−0.2987^***^ (−0.3823, −0.2150)	−0.2918^***^ (−0.3606, −0.2230)	−25.31%
TCM expenditures	990.97	714.60	−27.89%	720.25	454.56	−36.89%	0.7620^***^ (0.5399, 0.9841)	0.7322^***^ (0.5169, 0.9476)	107.97%

(1) We used Ordinary Least Square with robust standard errors in DID regression. (2) The unadjusted regression model only included the indicators of time and policy, and the interaction of time and policy. The regression model adjusted for participant characteristics and time trend variables, including age, gender, metastasis, treatment type, payment type, and length of stay. (3) The outcomes of expenditures in DID regression were transformed to logarithm, so the policy effects could be calculated by 100(e^β^-1) %.

(4) ^*^p < 0.05,

*** p < 0.001.

### Impact of the NCDP policy on health expenditures for lung cancer patients

The last three columns of Table 3 show the DID estimations and the policy effects. The DID estimation coefficients of total expenditures and drug expenditures were significantly negative, indicating that the NCDP policy could significantly reduce the overall spending of lung cancer patients. After the implementation of NCDP policy, the total expenditures and drug expenditures decreased by 0.1523 and 0.2326 log points, respectively, that is, a −14.13% change in total expenditures and a −20.75% change in drug expenditures.

Meanwhile, the results also identified significant decreases in health service expenditures (−7.65%), diagnosis expenditures (−38.28%), and consumable material expenditures (−25.31%) after the NCDP policy. The decrease in health service expenditures was mainly attributable to the decline in general medical service fees and nursing fees (Supplementary Table S1). The decrease in diagnosis expenditures was mainly attributable to the decline in laboratory diagnosis fees and imaging diagnosis fees (Supplementary Table S2). In addition, despite the declining trends in TCM expenditures, we found that the DID estimation coefficient was significantly positive, indicating that the NCDP implementation was associated with a 107.97% increase in TCM expenditures.

### Common trends test for DID

**Figure 2:**
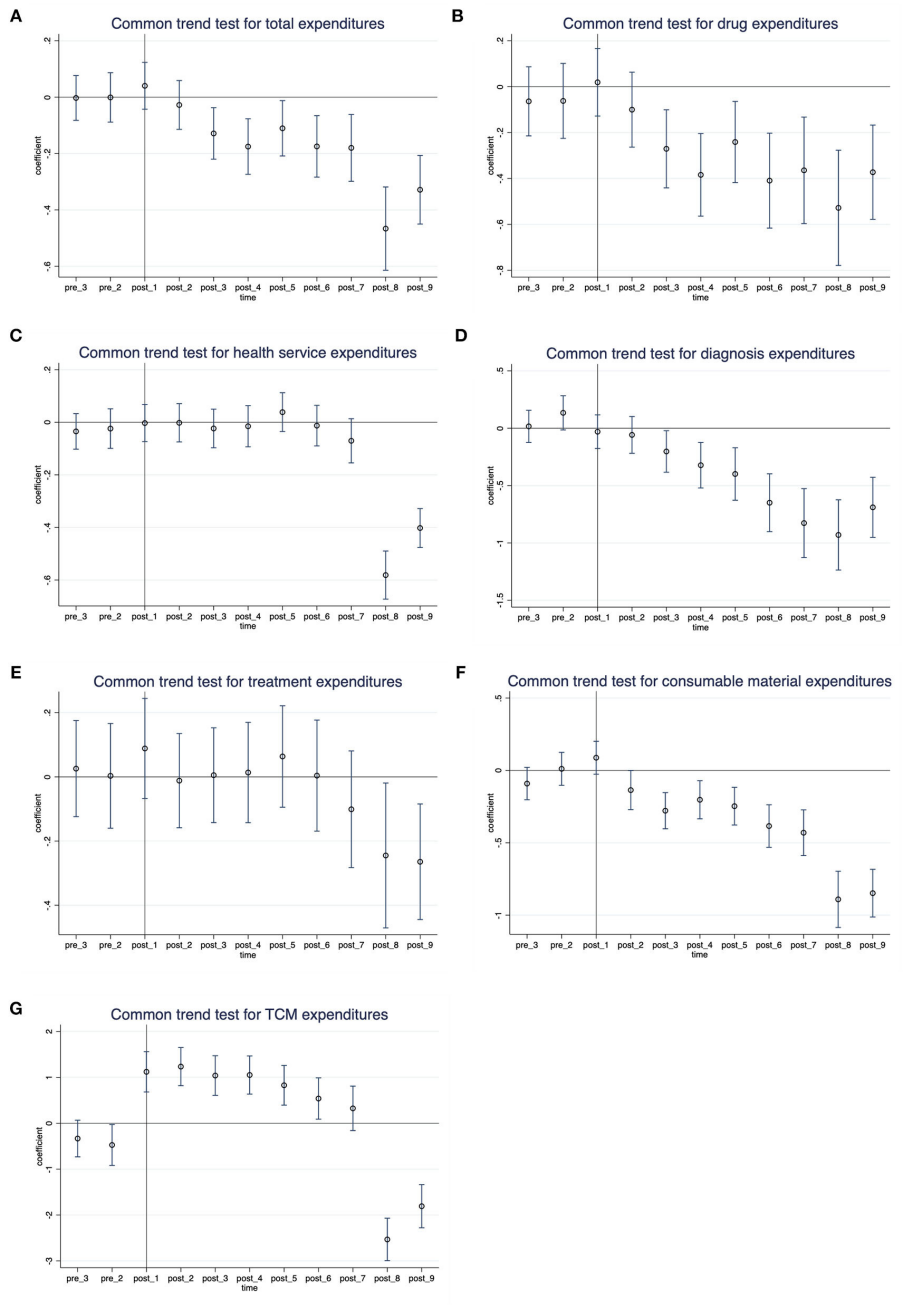
Common trends test for DID: Monthly differences between the treatment group and control group. **(A)** Total expenditures, **(B)** drug expenditures, **(C)** health service expenditures, **(D)** diagnosis expenditures, **(E)** treatment expenditures, **(F)** consumable material expenditures, and **(G)** TCM expenditures.

We tested for parallel trends by evaluating the differences in temporal trends between the intervention group and comparison group. Figure 2 shows the point estimates and 95% confidence interval (95% CI) of coefficients for the interaction variable between the month dummy variable and the intervention dummy variable. The coefficients of interaction before the policy were not significant (the 95% CI of coefficients contained zero), indicating that in the absence of the policy, the unobserved differences between the treatment group and the control group were the same over time. These results support that our identification strategy is appropriate.

### Sensitivity analyses

**Figure 3:**
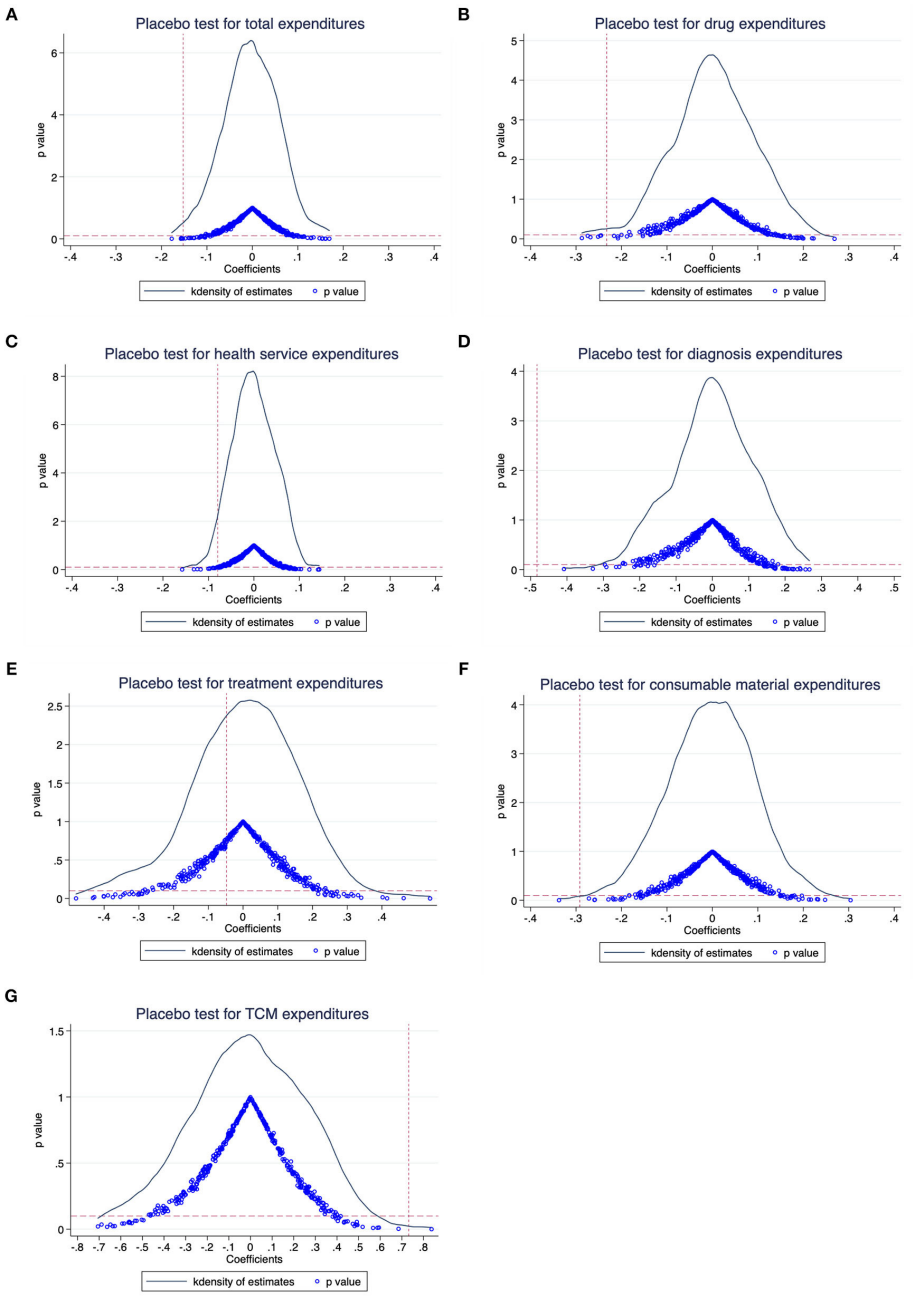
Placebo test results: The distribution diagrams of the coefficients. **(A)** Total expenditures, **(B)** drug expenditures, **(C)** health service expenditures, **(D)** diagnosis expenditures, **(E)** treatment expenditures, **(F)** consumable material expenditures, and **(G)** TCM expenditures.

We conducted four sensitivity analyses to prove that the main results are robust. First, we performed a placebo test to indirectly see whether the non-direct observable characteristics will affect the benchmark regression results (30). We kept the policy launched time and created a series of virtual treatment groups that were not affected by the policy. More specifically, the research sample in our main analysis contains 23,443 patients, of whom 3,636 patients were in treatment group. Therefore, we randomly selected 3,636 patients as a virtual treatment group, and the remaining 19,807 patients were used for a control group. Then, we conducted the DID regression as equation (1). This process was repeated 500 times and the distribution diagrams of the coefficients of DID are shown in Figure 3. The result found that the mean value of β was close to zero and significantly different from the actual regression coefficients (the red vertical dashed line), indicating that the main results were driven by the NCDP policy rather than other factors (e.g., the change of treatment patterns).

Second, we restricted our sample to avoid confounding by gender differences. Third, because all outcomes rapidly fell in November and rose in December, we excluded the patients in November and repeated the DID regression. Fourth, we used Fourier terms to control for the seasonality in study period. Other settings were the same as equation (1) in sensitivity analyses 2, 3 and 4, and the results are shown in Supplementary Tables S3–S5, which are similar to the main analysis.

## Discussion

### Interpretation of findings

To the best of our knowledge, this was the first study that investigated the effects of NCDP policy on hospitalization expenditures of cancer patients. We focused on cancer patients who were most likely affected by high medical costs. Our study showed that after the implementation of NCDP, the total expenditures and drug expenditures of hospitalization for lung cancer patients decreased by 14.13 and 20.75%, respectively. This result was consistent with previous studies and it was reasonable because the NCDP policy was expected to reduce pharmaceutical spending and improve the accessibility of medical services (21–23). The prices of the 25 winning drugs in the first round of NCDP dropped by an average of 52% with the highest drop of 96% (5). Such significant drug price reduction also affected the prices of non-selected original drugs (31). Eli Lilly, for example, the original development company of Pemetrexed, offered to reduce the price of Pemetrexed by 30% in some provinces.

The NCDP policy might have spillover effects on non-drug-related expenditures. Our study found significant decreases in health service expenditures, diagnosis expenditures, and consumable material expenditures, indicating that the patients' demand for medical services decreased during hospitalization. We figured out two possible explanations. First, gefitinib, one of the selected drugs, is an oral targeted anticancer drug used to treat NSCLC. Patients normally take oral anticancer drugs just at home thus reducing the length of hospital stays. As a result, demand of patients for treatment and healthcare in hospitals decreased after the NCDP. Second, the Chinese government launched the national performance appraisal of tertiary public hospitals since 2019 (29). A key indicator of income structure is the percentage of healthcare service income of total healthcare income, which is calculated by (healthcare service income/total income) ^*^100%. The healthcare service income is the total healthcare income except for drugs, consumables, and diagnostic income. This indicator is expected to be increased according to the document from the government (32). As the drug expenditures and total expenditures declined, physicians might decrease the use of diagnostic tests and consumable materials to make sure that the indicator was increased or stable. We calculated this percentage and showed the monthly trends in Supplementary Figure S1. The percentage of healthcare service income of total income for cancer patients was among 20%, and there was an increasing trend in the treatment group and a stable trend in the control group. Similar effects have been reported in previous studies. An earlier study about the Beijing Comprehensive Healthcare Reform found that after separating drug sales from hospital revenue, not only the drug costs but also the consumable costs were reduced (33). The spillover effects of NCDP could be considered to promote medical system reform in China in other aspects apart from drug bidding. We look forward to more studies to evaluate this effect.

However, this study found that the NCDP implementation was associated with a 107.97% increase in TCM expenditures. Similar results have been identified in other health policies in China. After the implementation of the National Essential Medicine System, many doctors reported that their fee-for-service activities increased, such as the prescribing of raw herbs and unprocessed traditional medicines (34). And the number of Western medicines per outpatient prescription decreased, while that of TCMs increased after the Drug Zero Mark-up policy (35). These studies suggested that physicians may increase prescriptions of TCM after such drug policies. A potential explanation is that the policy has an “income effect.” As the prices of drugs drop significantly, patients are more willing and affordable for these complementary and alternative treatments to relieve pain and improve the quality of life (36). In addition, some articles found that the purchase volume and expenditures of alternative drugs (which have an alternative relationship with the bid-winning products in clinical use) increased significantly after the implementation of NCDP policy (19, 20). Therefore, as the increase of TCM used could be related to several aspects, we could not give a specific reason for this effect, which should be considered in future studies.

### Policy implications

The study has several policy implications. First, this study provided evidence that the NCDP policy can indeed improve the affordability of selected drugs and reduce the financial burden on lung cancer patients. Until December 2021, six rounds of NCDP have been introduced, and the centralized procurement of high-value devices such as coronary stent, joint prosthesis, and intraocular lens has also been gradually carried out. More and more clinical necessary drugs and medical devices were included in the category of NCDP; thus, more patients could benefit from the policy. Second, after the implementation of NCDP, the health service expenditures, diagnosis expenditures, and consumable material expenditures of cancer inpatients have also been reduced, which indicated that the reform can promote the rational use of medical services and consumables in public hospitals. Third, we found that there was a significant increase in the use of alternative drugs after the policy. The policymakers should consider the related effects of health policy and monitor the utilization of both selected drugs and policy-related drugs to avoid the irrational use of such drugs (37, 38). Meanwhile, it is also necessary to promote the reform of the salary system in public hospitals and deal with the reliance on drug and consumable sales by increasing the income from health services.

### Strengths and limitations

Our study has a few strengths. First, our study used patient-level data to assess the effects of NCDP pilot program on lung cancer patients. To the best of our knowledge, this was the first study using patient discharge records to evaluate the impact of NCDP on various types of expenditures of patients, not only the drug expenditures but also other expenditures such as diagnosis expenditures and consumable material expenditures. The findings can comprehensively reflect the effects of NCDP on expenditures of patients during hospitalization. Second, we applied a DID study design to minimize the potential confounding in observational studies and improve the strength of findings. DID can remove bias in treatment effect estimation due to confounding by unobserved time-varying factors that have changed the outcome in treatment group and control group in the same way (25). We tested the assumption of parallel trends for DID analysis, and a series of sensitivity analyses were conducted to approve the robustness of main results.

There are some potential limitations in our study. First, the data was collected from a single healthcare institution, which may limit the generalizability of findings. However, it is the largest oncology specialized hospital in southwest China. Therefore, our sample is representative to evaluate the NCDP policy effects on cancer inpatients in pilot cities. Second, we included patients for only 1-year interval and focused on the first round of NCDP implemented in 2019. There were a series of policies focused on anticancer drugs in recent years, for example, the National Reimbursement Drug List was changed in January 2020 and the second round of NCDP was implemented in April 2020. In order to eliminate any possible confounding, we finally extracted the data from January 2019 to December 2019. Meanwhile, there were also some reforms during the study period, such as hospital vertical consolidation and prospective global budget, which may also explain the reduction in drug and non-drug costs among patients with cancer. Therefore, we used the DID method to minimize the impact of these reforms on our estimation. Third, information regarding oncological characteristics (stage and subtypes) were unavailable, for which we were unable to include more potential confounding. Finally, there were some potential reasons that might lead to the underestimation of policy effects. Our outcomes were the expenditures per hospitalization. But a patient might receive medication several times and seek services out of the hospital. Gefitinib is a common oral targeted therapy drug for NSCLC, and patients can buy and take it out of the hospital. The policy impact on actual spending of cancer patients might be larger than our estimation. Future studies should consider the whole economic burden for cancer patients using regional electronic medical record data.

## Conclusion

Using a DID design, we evaluated the impact of the pilot NCDP program on health expenditures of lung cancer inpatients. Our finding showed that the policy was associated with significant decreases in all types of expenditures, except for the TCM expenditures. Overall, the reform achieved its goals of high-quality and affordable healthcare. Further studies should assess the impact of NCDP on health outcomes, and consider the long-term effects of the drug procurement scheme.

## Data availability statement

The raw data supporting the conclusions of this article will be made available by the authors, without undue reservation.

## Author contributions

Conception or design of the work: S-yZ and XS. Data collection: S-yZ. Data analysis and interpretation: Y-jZ, YR, QZ, Y-xH, XZ, and KZ. Drafting the article: Y-jZ. Critical revision of the article: YR, JT, S-yZ, and XS. All authors have approved the final version of the manuscript.

## Funding

This study was supported by National Natural Science Foundation of China (Grant Nos. 72004149 and 71773080), Fundamental Research Funds for the Central public welfare research institutes (Grant No. 2020YJSZX-3), Sichuan Youth Science and Technology Innovation Research Team (Grant No. 2020JDTD0015), and 1·3·5 project for disciplines of excellence, West China Hospital, Sichuan University (Grant No. ZYYC08003).

## Conflict of interest

The authors declare that the research was conducted in the absence of any commercial or financial relationships that could be construed as a potential conflict of interest.

## Publisher's note

All claims expressed in this article are solely those of the authors and do not necessarily represent those of their affiliated organizations, or those of the publisher, the editors and the reviewers. Any product that may be evaluated in this article, or claim that may be made by its manufacturer, is not guaranteed or endorsed by the publisher.

## Abbreviations

NCDP: National Centralized Drug Procurement
DID: Difference-in-differences
NSCLC: Non-Small Cell Lung Cancer
TCM: traditional Chinese medicine
CNY: Chinese Yuan
CI: confidence interval.
