# Supplementary material for: The impact of national centralized drug procurement on health expenditures for lung cancer inpatients: A difference-in-differences analysis in a large tertiary hospital in China
Source: Front Public Health. 2022 Aug 12;10:956823. doi: 10.3389/fpubh.2022.956823 (PMC9412196; doi:10.3389/fpubh.2022.956823)
Supplement: Supplementary file 1 [file Data_Sheet_1.docx]

Supplementary Material

# Supplementary Tables

Supplementary Table 1. Impact of the NCDP policy on health service expenditures

|  | (1) | (2) | (3) | (4) |
| --- | --- | --- | --- | --- |
| VARIABLES | General medical service fees | Medical operation fees | Nursing fees | Other fees |
| Interaction | -0.120*** | -0.0663** | -0.116*** | 0.651*** |
|  | (0.0206) | (0.0273) | (0.0274) | (0.0646) |
| Time | 0.00668 | 0.187*** | 0.300*** | -1.184*** |
|  | (0.00749) | (0.0134) | (0.0104) | (0.0240) |
| Policy | 0.0701*** | 0.335*** | 0.409*** | -0.852*** |
|  | (0.0173) | (0.0243) | (0.0246) | (0.0591) |
| Constant | 5.142*** | 5.258*** | 4.835*** | 1.816*** |
|  | (0.0293) | (0.0407) | (0.0329) | (0.0711) |
| Covariates | Yes | Yes | Yes | Yes |
| Time trends | Yes | Yes | Yes | Yes |
| Observations | 23 443 | 23 443 | 23 443 | 23 443 |
| R-squared | 0.606 | 0.324 | 0.530 | 0.154 |

Notes: Robust standard errors in parentheses, *** p<0.01, ** p<0.05, * p<0.1.

Supplementary Table 2. Impact of the NCDP policy on diagnosis expenditures

|  | (1) | (2) | (3) | (4) |
| --- | --- | --- | --- | --- |
| VARIABLES | Pathological diagnosis fees | Laboratory diagnosis | Diagnostic imaging fees | Clinical diagnosis fees |
| Interaction | -0.0656 | -0.539*** | -0.335*** | -0.00247 |
|  | (0.0529) | (0.0457) | (0.104) | (0.0355) |
| Time | 0.00838 | 0.153*** | -0.0322 | -0.0248 |
|  | (0.0263) | (0.0182) | (0.0562) | (0.0163) |
| Policy | -0.00285 | 0.128*** | -0.0689 | -0.0719** |
|  | (0.0497) | (0.0322) | (0.0914) | (0.0334) |
| Constant | -0.398*** | 6.112*** | 4.991*** | -0.243*** |
|  | (0.0711) | (0.0561) | (0.144) | (0.0493) |
| Covariates | Yes | Yes | Yes | Yes |
| Time trends | Yes | Yes | Yes | Yes |
| Observations | 23 443 | 23 443 | 23 443 | 23 443 |
| R-squared | 0.044 | 0.121 | 0.065 | 0.058 |

Notes: Robust standard errors in parentheses, *** p<0.01, ** p<0.05, * p<0.1.

Supplementary Table 3. Sensitivity analysis 2 (exclude breast cancer, cervical cancer, ovarian cancer and corpus uteri cancer patients in the control group)

|  | (1) | (2) | (3) | (4) | (5) | (6) | (7) |
| --- | --- | --- | --- | --- | --- | --- | --- |
| VARIABLES | Total expenditures | Drug expenditures | Health service expenditures | Diagnosis expenditures | Treatment expenditures | Consumable material expenditures | TCM expenditures |
| Interaction | -0.274*** | -0.389*** | -0.181*** | -0.495*** | -0.240*** | -0.336*** | -0.602*** |
|  | (0.0282) | (0.0496) | (0.0237) | (0.0544) | (0.0656) | (0.0468) | (0.129) |
| Time | 0.0675*** | 0.0434 | 0.219*** | 0.132*** | 0.321*** | 0.0993** | 0.881*** |
|  | (0.0199) | (0.0293) | (0.0177) | (0.0390) | (0.0585) | (0.0405) | (0.0931) |
| Policy | 0.131*** | 0.100** | 0.363*** | 0.0152 | 0.744*** | 0.0156 | 1.347*** |
|  | (0.0235) | (0.0411) | (0.0210) | (0.0428) | (0.0581) | (0.0400) | (0.113) |
| Constant | 8.684*** | 8.011*** | 6.131*** | 6.564*** | 3.190*** | 5.416*** | 3.893*** |
|  | (0.0452) | (0.0687) | (0.0383) | (0.0877) | (0.114) | (0.0878) | (0.199) |
| Covariates | Yes | Yes | Yes | Yes | Yes | Yes | Yes |
| Time trends | Yes | Yes | Yes | Yes | Yes | Yes | Yes |
| Observations | 10 570 | 10 570 | 10 570 | 10 570 | 10 570 | 10 570 | 10 570 |
| R-squared | 0.299 | 0.165 | 0.573 | 0.206 | 0.204 | 0.222 | 0.129 |

Notes: Robust standard errors in parentheses, *** p<0.01, ** p<0.05, * p<0.1.

Supplementary Table 4. Sensitivity analysis 3 (exclude the patients in November 2019)

|  | (1) | (2) | (3) | (4) | (5) | (6) | (7) |
| --- | --- | --- | --- | --- | --- | --- | --- |
| VARIABLES | Total expenditures | Drug expenditures | Health service expenditures | Diagnosis expenditures | Treatment expenditures | Consumable material expenditures | TCM expenditures |
| Interaction | -0.142*** | -0.226*** | -0.0633*** | -0.443*** | -0.0799* | -0.263*** | 0.760*** |
|  | (0.0246) | (0.0459) | (0.0195) | (0.0473) | (0.0476) | (0.0352) | (0.111) |
| Time | -0.0544*** | -0.147*** | 0.109*** | 0.0516** | 0.229*** | -0.0366* | 0.0336 |
|  | (0.0111) | (0.0161) | (0.00835) | (0.0203) | (0.0306) | (0.0206) | (0.0536) |
| Policy | 0.0274 | -0.0534 | 0.252*** | 0.00304 | 0.477*** | 0.0605** | 0.279*** |
|  | (0.0202) | (0.0376) | (0.0173) | (0.0342) | (0.0435) | (0.0304) | (0.0981) |
| Constant | 8.867*** | 8.235*** | 6.266*** | 6.788*** | 3.336*** | 5.768*** | 5.366*** |
|  | (0.0339) | (0.0499) | (0.0291) | (0.0639) | (0.0849) | (0.0648) | (0.160) |
| Covariates | Yes | Yes | Yes | Yes | Yes | Yes | Yes |
| Time trends | Yes | Yes | Yes | Yes | Yes | Yes | Yes |
| Observations | 21 580 | 21 580 | 21 580 | 21 580 | 21 580 | 21 580 | 21 580 |
| R-squared | 0.283 | 0.140 | 0.577 | 0.148 | 0.144 | 0.213 | 0.190 |

Notes: Robust standard errors in parentheses, *** p<0.01, ** p<0.05, * p<0.1.

Supplementary Table 5. Sensitivity analysis 4 (control for the potential seasonality)

|  | (1) | (2) | (3) | (4) | (5) | (6) | (7) |
| --- | --- | --- | --- | --- | --- | --- | --- |
| VARIABLES | Total expenditures | Drug expenditures | Health service expenditures | Diagnosis expenditures | Treatment expenditures | Consumable material expenditures | TCM expenditures |
| Interaction | -0.151*** | -0.231*** | -0.0799*** | -0.484*** | -0.0488 | -0.291*** | 0.733*** |
|  | (0.0246) | (0.0456) | (0.0193) | (0.0471) | (0.0477) | (0.0350) | (0.110) |
| Time | -0.0344 | -0.0328 | -0.00986 | 0.0247 | 0.141* | 0.0480 | -0.327*** |
|  | (0.0280) | (0.0424) | (0.0207) | (0.0514) | (0.0726) | (0.0475) | (0.126) |
| Policy | 0.0233 | -0.0554 | 0.250*** | 0.000997 | 0.466*** | 0.0644** | 0.229** |
|  | (0.0202) | (0.0376) | (0.0173) | (0.0343) | (0.0436) | (0.0302) | (0.0980) |
| Constant | 8.842*** | 8.193*** | 6.212*** | 6.710*** | 3.249*** | 5.657*** | 4.675*** |
|  | (0.0389) | (0.0578) | (0.0311) | (0.0732) | (0.0989) | (0.0726) | (0.174) |
| Covariates | Yes | Yes | Yes | Yes | Yes | Yes | Yes |
| Time trends | Yes | Yes | Yes | Yes | Yes | Yes | Yes |
| Seasonality | Yes | Yes | Yes | Yes | Yes | Yes | Yes |
| Observations | 23,443 | 23,443 | 23,443 | 23,443 | 23,443 | 23,443 | 23,443 |
| R-squared | 0.292 | 0.150 | 0.600 | 0.153 | 0.155 | 0.221 | 0.251 |

Notes: Robust standard errors in parentheses, *** p<0.01, ** p<0.05, * p<0.1.

Supplementary Figure 1. The percentage of healthcare service income of total healthcare income (calculate by (healthcare service income/total income) *100%)

Notes: The healthcare service income is the total healthcare income expect for drugs, consumables, and diagnostic income.
